# Critical Nutrients in the Ketogenic Diet for Adolescents Based on Optimized Hypothetical Meal Plans

**DOI:** 10.3390/nu18132101

**Published:** 2026-06-27

**Authors:** Marc Assmann, Isabel Albrecht, Tobias Fischer

**Affiliations:** Center for Nutrition and Therapy (NuT), University of Applied Sciences Muenster, Corrensstraße 25, 48149 Muenster, Germany

**Keywords:** ketogenic diet, ketogenic metabolic therapy, adolescents, micronutrients, critical nutrients, optimized meal plans

## Abstract

**Background:** Ketogenic diets are used as a non-pharmacological treatment for drug-resistant epilepsy in childhood and adolescence. However, the potential micronutrient deficiencies associated with ketogenic diets have not been adequately investigated in vulnerable groups, such as children and adolescents, and detailed dietary analyses are lacking. **Methodology:** Optimized ketogenic daily meal plans were created for adolescents aged 10–18 years with different ketogenic ratios of 3:1, 2:1, and 1:1. Micronutrient supply was calculated using PRODI^®^ nutrition software (version 7.3, Nutri-Science GmbH, Freiburg, Germany), based on the German Nutrient Database and compared with DGE/ÖGE reference values. Nutrients below 95% of the reference values were classified as potentially critical. **Results:** The results showed that micronutrient density decreased with increasing dietary restriction. Vitamin D and fiber were below reference values for all ratios and age groups. The 3:1 ratio exhibited deficiencies in potassium, zinc, fluoride, and several B vitamins. Overall, the 1:1 ratio provided the most favorable nutrient coverage, though vitamin B_1_ and fluoride remained insufficient. The results indicate that potentially critical micronutrients are highly sensitive to ketogenic ratios in adolescents. **Conclusions:** This analysis enables the identification of relevant nutrients to be more targeted, suggesting that a one-size-fits-all approach of supplementation should be replaced by options differentiated by age and dietary restrictions.

## 1. Introduction

Nationwide, representative nutritional data from healthy children and adolescents aged six to seventeen across Germany showed that even those on a mixed diet do not consume a sufficient amount of several micronutrients. Notably, deficiencies in vitamin D, folate, calcium, iodine, and potassium were observed across all age groups [1]. Against this background, special dietary requirements are coming into focus. Due to their restrictive nature, these requirements may pose a potential further risk of an insufficient nutrient supply. One such diet is the ketogenic diet (KD), which national and international guidelines and recommendations establish as an option for treating drug-resistant epilepsy in children and adolescents [2,3]. The KD is high in fat, very low in carbohydrates, and moderate in protein. During fasting or when carbohydrate intake is greatly reduced, as with the KD, blood glucose levels drop, inducing ketogenesis. This increases the production of ketone bodies, primarily β-hydroxybutyrate and acetoacetate, in the liver, which serve as an energy source. A shift in energy metabolism toward fatty acid oxidation results in an increased concentration of ketone bodies in the blood (ketosis) and a reduced glycogenic energy supply [4,5,6]. Because of the restriction of carbohydrate-rich foods in KD, key food groups highlighted in national and international dietary guidelines, such as grain products and fruits, are eliminated. However, these food groups are important sources of micronutrients in a mixed diet. Reducing the number of food groups considerably increases the risk of exacerbating existing nutritional deficits [1,7].

Originally developed in the 1920s, the KD was intended as a non-pharmacological therapy for epilepsy [8]. Today, in addition to epilepsy, the KD is used to treat rare inherited metabolic disorders, such as glucose transporter 1 deficiency syndrome (GLUT1-DS) and pyruvate dehydrogenase (PDH) deficiency [2,3,9]. The composition of KD is often defined by the ketogenic ratio, which is the ratio of fat to the sum of carbohydrates and proteins. In classic KD (cKD), the ratio is 4:1 or 3:1; however, in practice, less restrictive variants below 3:1 are also used. These ratios allow for a higher protein intake, which increases the diet’s suitability for everyday use [2,4]. Studies show that different ratios can be equally effective in controlling seizures, and more liberal variants, such as the modified Atkins diet (MAD), are often better tolerated and associated with improved micronutrient density [10,11]. An analysis of modeled nutrition plans revealed that 12 of the 28 examined nutrients met the Dietary Reference Intakes (DRIs) for a KD ratio of 1:1, whereas only 3 met the DRIs for a KD ratio of 4:1 [12]. The high fat content (60 to 90 percent of energy, E%) significantly limits the selection of micronutrient-rich foods and can therefore impair micronutrient supply. Further studies based on dietary records and plasma micronutrient analyses indicate lower micronutrient density in KD compared to mixed diets. These studies describe deficiencies in nutrients, such as vitamin D, calcium, iron, zinc, and folate. Accordingly, regular check-ups and targeted supplementation are recommended [2,13]. Due to the limitations in food choice and potentially reduced micronutrient density of KDs, ensuring an adequate micronutrient supply is crucial, especially for children and adolescents. Data on micronutrient supply under KDs for the 10- to 18-year-old age group is very limited. This life stage is characterized by accelerated growth, hormonal changes, and increased energy and nutrient requirements, which increases the risk of nutrient undersupply [14].

In addition to nutritional considerations, practical implementation of the KD poses challenges, particularly for children and adolescents [15,16]. Social restrictions, school meals, leisure-time meals, the loss of autonomy associated with the diet, and the high preparation costs make consistent adherence difficult [17]. Studies also report low compliance rates, often attributing discontinuation to side effects, psychosocial stress, or the restrictive nature of the diet [18,19].

Due to the limited available data, an analysis of hypothetical ketogenic daily meal plans for adolescents aged 10 to 18 years in the ratios of 3:1, 2:1, and 1:1 were performed. While previous studies have primarily assessed micronutrient intake based on dietary records or clinical cohorts, it remains unclear whether reported inadequacies are mainly due to suboptimal dietary implementation or reflect the inherent nutritional challenges of ketogenic diets. The hypothetical meal plans were compared with the national reference values of the German Nutrition Society (DGE) and the Austrian Nutrition Society (ÖGE) (DGE/ÖGE reference values) to identify nutrients that may be difficult to provide in adequate amounts, even under carefully planned dietary conditions. The objective was to expand the available evidence on the nutritional adequacy of ketogenic diets, identify critical nutrients, and derive practical conclusions for planning KDs for adolescents.

## 2. Materials and Methods

### 2.1. Data Acquisition

To investigate nutrient coverage at different ketogenic ratios, optimized, hypothetical daily ketogenic meal plans were created for males aged 10 to 18 at ketogenic ratios of 3:1, 2:1, and 1:1. Three age groups were considered, according to the DGE/ÖGE reference values: 10–12, 13–14, and 15–18 years [20]. A total of three daily plans were developed for each age group and ratio (*N* = 27).

The daily meal plans were created using PRODI Expert Nutrition Software (version 7.3; Nutri-Science GmbH, Freiburg, Germany) based on foods and nutritional values from the German Nutrition Database (BLS, version 3.02). First, nutrition professionals created three meal plans for each age group at a ratio of 3:1. These initial meal plans were made using commonly consumed omnivorous foods suitable for ketogenic diets. The meal composition was based on existing ketogenic recipes and practical meal examples from clinical and educational resources. Food selection was not restricted to predefined lists, but rather was based on professional judgment to create realistic meal plans that maximize nutrient coverage within the predefined dietary constraints. The 3:1 meal plans then formed the basis for the development of meal plans with ratios of 2:1 and 1:1. The 2:1 and 1:1 meal plans were generated by adjusting the proportions of foods containing fat, protein and carbohydrates, while keeping the structure of the meals the same. Energy supply was based on DGE/ÖGE reference values: 2200 kcal for 10–12-year-olds, 2600 kcal for 13–14-year-olds, and 3000 kcal for 15–18-year-olds. The calculation was performed using a PAL (physical activity level) of 1.6, with a permissible deviation of ±5% from the respective age-related reference energy. The amount of fat was adjusted so that the desired ketogenic ratio per meal deviates by no more than ±0.5 from the target ratio (3:1, 2:1, or 1:1). For the 3:1 ratio, the maximum carbohydrate supply was set at 50 g/day, and for the 2:1 and 1:1 ratios, the upper limit was set at ≤60 g/day in accordance with a low glycemic index treatment (LGIT) [2]. For the 3:1 and 2:1 ratios, the amount of protein was calculated based on the age-adjusted DGE/ÖGE reference values (target range: ±5%). For the 1:1 ratio, the absolute amount of protein was defined according to the LGIT (≤30 percent of daily energy; E%) [2]. Each daily meal plan included three main meals (breakfast, lunch, and dinner) and two snacks to ensure an even distribution of nutrients and energy throughout the day in accordance with the German guidelines for ketogenic nutritional therapies [3]. Water supply was calculated using the age-adjusted DGE/ÖGE reference values for total water intake.

After exporting the data records to Microsoft Excel (version 2510), the database was reviewed and standardized. Equivalent products from the BLS database (version 3.02) were used to replace foods whose nutritional values were not based on BLS data, or that contained incomplete or no nutritional information. Furthermore, the fiber content and amounts of all vitamins and minerals for which DGE/ÖGE reference values were available were calculated for each daily meal plan based on BLS data. Minerals without sufficient BLS data (chromium, selenium, and molybdenum) were not considered.

### 2.2. Statistical Analysis

Fiber, vitamin, and mineral coverage was presented as a percentage of the respective reference values (target range: ≥95%). Falling below the 95% limit of a reference value was classified as potentially critical and falling below the 50% limit was classified as significantly deficient. Percentage deviations from reference values, mean values, standard deviations, and ranges were calculated using Microsoft Excel.

## 3. Results

### 3.1. Achieving Energy and Macronutrient Goals

The average energy supply was 2124 ± 42 kcal for the 10–12 age group, 2530 ± 74 kcal for the 13–14 age group, and 2933 ± 113 kcal for the 15–18 age group. These values correspond to 96–98% of the reference values (target range: 95–105%). Across all age groups, carbohydrate supply was 34.1 ± 5.7 g/day for a 3:1 ratio, 54.5 ± 3.7 g/day for a 2:1 ratio, and 58.8 ± 0.9 g/day for a 1:1 ratio. These values were all below the upper limits of 50 and 60 g/day, respectively. The fat content decreased from 88.3 ± 0.6 E% (3:1 ratio), to 83.7 ± 0.6 E% (2:1 ratio) and then to 76.3 ± 0.4 E% (1:1 ratio). Protein amounts were 0.91 ± 0.03 g/kg body weight (BW) for the 3:1 ratio, 0.93 ± 0.02 g/kg BW for the 2:1 ratio, and 1.63 ± 0.04 g/kg BW for the 1:1 ratio.

### 3.2. The Nutrient Coverage Compared to Age-Specific Reference Values

**Figure 1:**
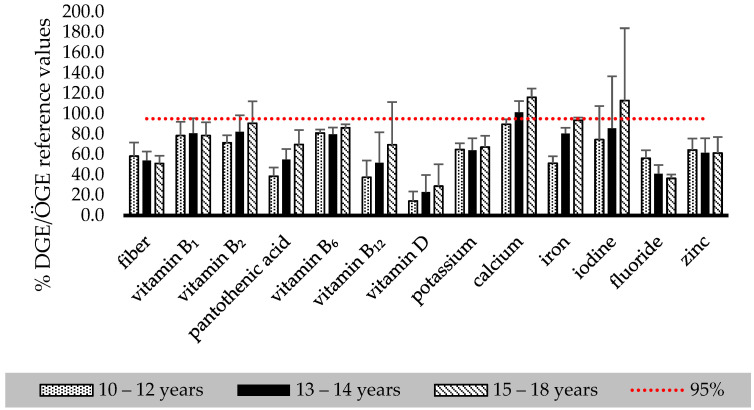
Percentage fulfillment of the analyzed DGE/ÖGE reference values at a 3:1 ratio, achieving an average of less than 95% of the reference values in at least one age group. The figure is divided into the following age groups: 10–12 years, 13–14 years, and 15–18 years, in accordance with the DGE/ÖGE reference values.

At a ratio of 3:1, thirteen nutrients were below 95% of the reference values in the 10–12 age group, twelve in the 13–14 age group, and eleven in the 15–18 age group (see Figure 1). The following nutrients were below 95% of the reference values for all age groups at a 3:1 ratio: fiber (51–58%), vitamin B_1_ (78–81%), vitamin B_2_ (71–91%), pantothenic acid (38–70%), vitamin B_6_ (80–86%), vitamin B_12_ (37–69%), vitamin D (14–29%), potassium (64–67%), calcium (90–116%), iron (51–93%), iodine (74–113%), fluoride (36–56%), and zinc (61–64%) (see Table 1).

**Figure 2:**
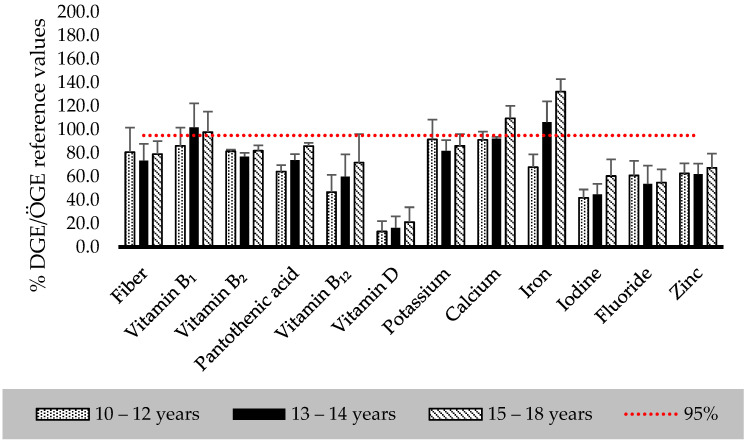
Percentage fulfillment of the analyzed DGE/ÖGE reference values at a 2:1 ratio, achieving an average of less than 95% of the reference values in at least one age group. The figure is divided into the following age groups: 10–12 years, 13–14 years, and 15–18 years, in accordance with the DGE/ÖGE reference values.

At a 2:1 ratio, the number of nutrients below 95% of the reference values was as follows: twelve in the 10–12 age group, ten in the 13–14 age group, and nine in the 15–18 age group (Figure 2). The analysis for all age groups indicated that the following nutrients were potentially critical for the 2:1 ratio: fiber (74–81%), vitamin B_1_ (86–102%), vitamin B_2_ (77–82%), pantothenic acid (64–86%), vitamin B_12_ (47–72%), vitamin D (13–21%), potassium (82–92%), calcium (91–110%), iron (68–132%), iodine (42–61%), fluoride (54–61%), and zinc (62–67%) (see Table 1).

**Figure 3:**
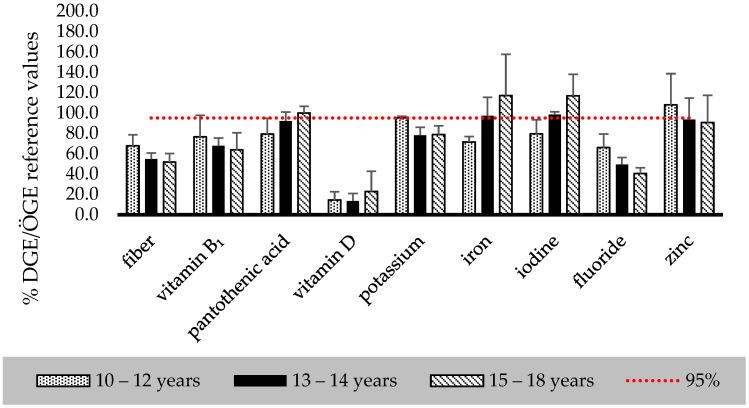
Percentage fulfillment of the analyzed DGE/ÖGE reference values at a 1:1 ratio, achieving an average of less than 95% of the reference values in at least one age group. The figure is divided into the following age groups 10–12 years, 13–14 years, and 15–18 years, in accordance with the DGE/ÖGE reference values.

At a 1:1 ratio, seven nutrients were below 95% of the reference values for the 10–12 and 13–14 age groups, while six nutrients fell below this threshold for the 15–18 age group (see Figure 3). The nutrients identified as potentially critical across all age groups at this ratio were fiber (52–68%), vitamin B_1_ (64–77%), pantothenic acid (79–100%), vitamin D (13–23%), potassium (78–95%), iron (71–117%), iodine (79–117%), fluoride (41–66%), and zinc (91–108%) (see Table 1).

### 3.3. Undershooting of Threshold Values Meal Plans Dependent on the Ratio

At a 3:1 ratio, all daily meal plans for all age groups were below the 95% threshold for eight nutrients. Five of these nutrients also had several daily meal plans with values below the 50% threshold. These were fiber (4/9 daily meal plans, 44%), pantothenic acid (4/9 daily meal plans, 44%), vitamin D (9/9 daily meal plans, 100%), fluoride (6/9 daily meal plans, 67%), and zinc (2/9 daily meal plans, 22%). By contrast, all daily meal plans for six nutrients were below the 95% threshold in the 2:1 ratio. Of these six nutrients, only three were below the 50% threshold in several meal plans. Specifically, these were vitamin B_12_ (4/9 daily meal plans, 44%), vitamin D (9/9 daily meal plans, 100%), and fluoride (4/9 daily meal plans, 44%). Furthermore, the 1:1 ratio showed a further decrease in critical nutrients across all daily meal plans. Overall, all daily meal plans were below the 95% threshold for three nutrients. All three had several meal plans below the 50% threshold for fiber (3/9 daily meal plans, 33%), vitamin D (9/9 daily meal plans, 100%), and fluoride (5/9 daily meal plans, 56%). The results are shown in Table 2.

## 4. Discussion

Overall, the present analysis showed that stricter ratios may be associated with lower micronutrient density in adolescents. However, the 1:1 ratio therefore achieved the highest coverage of reference values in the hypothetical, optimized meal plans. This finding is consistent with previous research on the KD [12,21,22]. Unlike previous studies based primarily on dietary records, the current modeling approach indicates that dietary restriction itself can impact the ability to meet micronutrient reference values, even under carefully planned dietary conditions.

The hypothetical nutrition meal plans presented here had a fiber supply of 50–80% of the DGE/ÖGE reference values for all age groups and ketogenic ratios [23]. These results are consistent with the finding of insufficient fiber intake in the context of a KD, which is primarily limited by severe restrictions on carbohydrate- and fiber-rich foods [2,24]. Nevertheless, studies by Volpe et al. (2007) [25] on children with drug-resistant epilepsy revealed that fiber intake can significantly decrease even before initiating a KD compared to that of healthy peers. This was attributed to restricted food intake due to frequent seizures and side effects of antiepileptic medication [25]. However, recent analyses of less restrictive KDs indicate that sufficient fiber intake can be achieved by specifically integrating fiber-rich, low-carbohydrate foods into the daily diet. For the MAD in particular, studies have shown that using vegetables, seeds, fiber-enriched products, and functional ingredients can help meet reference values more effectively [22]. The present analysis corroborates these findings, demonstrating that fiber intake remained below the reference value in most of the optimized meal plans despite targeted dietary planning. This suggests that providing adequate fiber may be difficult, especially in more restrictive ketogenic approaches.

The low coverage of vitamin D and calcium in stricter ratios is consistent with previous studies documenting insufficient intake of these nutrients in children and adolescents under KD, as determined by dietary protocols, serum micronutrient analyses, and clinical observations of bone health [2,14,26]. A recent review on the importance of vitamin D metabolism in KD confirms the relevance of vitamin D [27]. These findings are particularly important in light of growth and bone health during adolescence. This life period is characterized by rapid skeletal growth and the development of peak bone mass. Therefore, adequate vitamin D and calcium intake is essential to support bone mineralization and normal skeletal development. Inadequate intake of these nutrients during this developmental period may compromise optimal bone accretion and increase the risk of impaired bone health later in life [28,29]. However, inadequate vitamin D intake is not limited to KDs. Population-based data show that children, adolescents, and adults in Germany are significantly below the national reference values for vitamin D, regardless of their diet. This undersupply is primarily due to the low natural vitamin D density of common foods, limited intake via fortified products, and insufficient endogenous synthesis resulting from limited UV-B exposure [1,30]. Accordingly, vitamin D is not classified as a diet-specific risk nutrient in ketogenic nutrition guidelines, but rather as a general critical nutrient that may require routine supplementation, even with a mixed diet [2]. Regarding calcium, it should be noted that calcium levels in the hypothetical meal plans were slightly below the reference values in younger age groups with higher ketogenic ratios. Therefore, the data indicated a higher calcium supply than that reported in the literature [22,24,25], which may be attributed to the exclusive use of calcium-rich mineral water (>150 mg calcium per liter) as a beverage source, among other factors. This observation demonstrates how targeted food selection can compensate for potential inadequacies and underscores the importance of dietary planning in ketogenic nutritional therapy.

The present evaluation revealed critical supply patterns for B vitamins. These results are consistent with literature describing an insufficient supply of several B vitamins, particularly B_1_, B_2_, and B_6_, based on dietary protocols and nutrient calculations in pediatric ketogenic nutritional therapy [21,31]. Interestingly, pantothenic acid and vitamin B_12_ showed low coverage in several hypothetical meal plans, especially at higher ketogenic ratios. Unlike vitamins B_1_, B_2_, and B_6_, these vitamins have received comparatively little attention in the literature on the KD and may warrant further investigation in future studies. One possible explanation for the low vitamin B_12_ coverage is that major dietary sources of vitamin B_12_, such as meat, fish, eggs, and dairy products, also contribute substantial amounts of protein. In the present meal plans, protein intake was limited by the predefined ketogenic targets, especially at higher ratios. Consequently, increasing vitamin B_12_ intake through larger amounts of animal-derived foods was only possible to a limited extent. However, further analyses show that more liberal ketogenic approaches, such as the MAD and the use of multivitamin supplements, can significantly improve B vitamin intake [22]. Guidelines recommend micronutrient supplementation as an essential component of ketogenic therapy, explicitly emphasizing the relevance of B vitamins [2,12]. A comparison of evaluations with and without supplements, such as the present meal plan analysis, is not meaningful.

In addition, insufficient coverage of reference values was observed for the minerals iron, potassium, iodine, fluoride, and zinc, with increasing restrictiveness. The shortfall was consistent for potassium, which was below the reference values in nearly all ratios and age groups, which is consistent with findings of other researchers. Analyses based on dietary records repeatedly describe potassium as a critical nutrient that is not reliably met, even under more liberal ketogenic variants [21,22,31]. Fluoride was below the reference values in all examined ratios and age groups, a finding not mentioned in previous analyses. In contrast, iron and zinc were predominantly insufficient. Based on dietary records, repeated deficiencies in both trace elements have been described under cKD [22,31]. Adequate iron intake is especially important during adolescence because of increased needs related to rapid growth, increased blood volume, and increased lean body mass. For adolescent girls, iron requirements increase further following the onset of menstruation. Zinc is also essential during this stage of life because of its role in numerous physiological functions and its importance for normal growth and tissue development [32,33]. However, higher intakes were already achieved with less restrictive variants, such as MAD. Nevertheless, these still do not reliably meet the requirements of all age groups [21,22,31]. In this analysis, iodine was also below the reference values depending on the ratio and age group. Corresponding data from recent studies reported both insufficient and adequate intakes of iodine, depending on the diet, indicating high sensitivity to specific food choices [22,31]. Overall, the present results confirm previous observations from dietary records and clinical studies. The results also indicate that it may be difficult to provide adequate amounts of several minerals and trace elements even under optimized dietary conditions. Therefore, these findings suggest that some of the observed inadequacies may be due to the nutritional challenges posed by KD patterns themselves, rather than the manner in which the diet is implemented. While these findings are based on optimized hypothetical meal plans rather than dietary intake, they may help to identify nutrients that are difficult to obtain in adequate amounts through ketogenic diets for children and adolescents.

It should be noted that the daily meal plans presented are based on male reference values. For female adolescents, the age-dependent reference values for energy intake are, on average, 9% (ages 10–12), 15% (ages 13–14), and 24% (ages 15–18) lower than the corresponding values for males [20]. Several of the identified critical micronutrients, such as pantothenic acid, vitamin B_12_, potassium, calcium, iron (ages 10–12), and iodine have gender-independent reference values. For iron, the recommended intake for females aged 13–18 is around 50% higher [34]. Therefore, with lower energy requirements, females would need higher nutrient intake, i.e., higher micronutrient density, to reach these reference values. This further exacerbates the potentially inadequate supply of these micronutrients.

The used hypothetical optimized ketogenic meal plans represent an idealized implementation of the respective ratios. It does not take into account individual variability in eating habits, different food preferences, daily variations, or omissions of individual meals. Nevertheless, the optimized structure enables the identification of systematic, ratio-dependent nutrient supply patterns that may exist independently of individual consumption habits. This is a key strength of the modeling approach because it allows for the evaluation of theoretical nutrient adequacy under controlled conditions. It may also help distinguish between implementation-related and diet-related nutritional challenges. Another limitation is based on the characteristics of the nutritional database (BLS 3.02) used. There are no data sets available for chromium, selenium, and molybdenum; therefore, these micronutrients could not be included in the analysis. Additionally, the average values stored in the database only reflect real product variations, fortifications, and processing differences only to a limited extent.

While the results do not allow conclusions about the development of actual nutrient status, biochemical deficiencies, or clinical outcomes, they do indicate micronutrients that require special attention in the context of KD therapy. Therefore, the present findings should be interpreted as an assessment of theoretical nutrient adequacy under optimized dietary conditions, rather than as evidence of deficiencies that may occur in clinical practice. Further research on real-life consumption scenarios and the efficacy of supplementation strategies is necessary to identify critical nutrients in everyday clinical practice. Previous studies of children and adolescents on KDs showed that, even with adequate supplementation, growth and bone health may be affected [14,35]. This underscores the importance of continuous nutritional therapy support.

Clinical guidelines usually recommend standardized supplementation to compensate for potential deficiencies in practical implementation [2]. However, recent studies indicate that individual micronutrients such as zinc, vitamin A, and selenium may exceed the recommended upper intake limits, especially in more liberal KDs and combined supplementation [22]. These findings may provide a base for supplementation strategies with regard to both undersupply and possible oversupply by identifying nutrients requiring particular attention during dietary planning and monitoring.

## 5. Conclusions

The analysis of hypothetical optimized meal plans showed that the amount of nutrients in KDs can vary among adolescents, depending on the ratio. While some deficiencies were observed, the 1:1 ratio exhibited the highest overall nutrient values among the examined ketogenic ratios, whereas the 2:1 and 3:1 ratio demonstrated the most potential deficiency profiles. Possible nutrient deficits apply to well-known critical nutrients such as vitamin D, calcium, vitamin B_1_, and fluoride. Although the results represent hypothetical, optimized dietary meal plans for males with a PAL of 1.6, they highlight the importance of ensuring an adequate supply of micronutrients when implementing KDs in practice. However, rather than providing basic supplementation to every individual, a differentiated, ratio-sensitive supplementation strategy combined with nutritional therapy support should be adopted.

## Figures and Tables

**Table 1:** Percentage fulfillment and standard deviation (SD) of the analyzed DGE/ÖGE reference values across all age groups where the values fell below 95% of the reference values in at least one group (see Figure 1, Figure 2 and Figure 3).

Nutrient	3:1 (Mean in % ± SD)	2:1 (Mean in % ± SD)	1:1 (Mean in % ± SD)
fiber	54.4 ± 9.4	77.8 ± 14.2	58.0 ± 10.5
vitamin B_1_	79.1 ± 12.2	95.2 ± 17.2	69.2 ± 15.2
vitamin B_2_	81.3 ± 16.3	80.1 ± 3.8	138.3 ± 9.7
pantothenic acid	54.3 ± 16.7	74.7 ± 10.2	90.4 ± 13.2
vitamin B_6_	82.3 ± 5.0	108.8 ± 14.9	109.7 ± 17.3
vitamin B_12_	52.7 ± 30.6	59.5 ± 20.2	218.5 ± 75.4
vitamin D	21.9 ± 15.7	16.9 ± 9.8	16.8 ± 12.3
potassium	65.3 ± 8.8	86.5 ± 11.7	84.0 ± 10.2
calcium	102.3 ± 13.7	97.6 ± 11.1	130.2 ± 16.8
iron	75.0 ± 19.3	102.2 ± 30.4	95.2 ± 29.9
iodine	91.0 ± 49.8	49.0 ± 12.6	98.2 ± 20.6
fluoride	44.5 ± 10.8	56.4 ± 12.0	51.9 ± 13.8
zinc	62.3 ± 12.2	64.0 ± 9.0	97.3 ± 24.3

**Table 2:** Proportion of daily meal plans falling below reference values (less than 50% or 50–95%) across all age groups sorted by nutrient and ratio (*n* = 9).

Nutrient	3:1 < 50%, *n* (%)	3:1 50 < 95%, *n* (%)	2:1 < 50%, *n* (%)	2:1 50 < 95%, *n* (%)	1:1 < 50%, *n* (%)	1:1 50 < 95%, *n* (%)
fiber	4 (44)	5 (56)	0 (0)	8 (89)	3 (33)	6 (67)
vitamin B_1_	0 (0)	9 (100)	0 (0)	4 (44)	1 (11)	7 (78)
vitamin B_2_	0 (0)	6 (67)	0 (0)	9 (100)	0 (0)	0 (0)
pantothenic acid	4 (44)	5 (56)	0 (0)	9 (100)	0 (0)	4 (44)
vitamin B_6_	0 (0)	9 (100)	0 (0)	2 (22)	0 (0)	2 (22)
vitamin B_12_	4 (44)	4 (44)	4 (44)	5 (56)	0 (0)	0 (0)
vitamin D	9 (100)	0 (0)	9 (100)	0 (0)	9 (100)	0 (0)
potassium	0 (0)	9 (100)	0 (0)	7 (78)	0 (0)	8 (89)
calcium	0 (0)	3 (33)	0 (0)	5 (56)	0 (0)	0 (0)
iron	2 (22)	6 (67)	0 (0)	4 (44)	0 (0)	5 (56)
iodine	1 (11)	5 (56)	0 (0)	4 (44)	0 (0)	3 (33)
fluoride	6 (67)	3 (33)	4 (44)	5 (56)	5 (56)	4 (44)
zinc	2 (22)	7 (78)	0 (0)	9 (100)	0 (0)	5 (56)

## Data Availability

The data presented in this study are available on request from the corresponding author due to the continuous extension of the meal plan database.

## Abbreviations

The following abbreviations are used in this manuscript:

BLS	German Nutrition Database
BW	Body weight
cKD	Classic KD
DGE	German Nutrition Society
DRIs	Dietary Reference Intakes
E%	Percent of energy
GLUT1-DS	Glucose transporter type 1 deficiency syndrome
KD	Ketogenic diet
LGIT	Low glycemic index
MAD	Modified Atkins diet
ÖGE	Austrian Nutrition Society
PAL	Physical activity level
PDH	Pyruvate dehydrogenase
SD	Standard deviation
